# Flow Control in Wells Turbines for Harnessing Maximum Wave Power

**DOI:** 10.3390/s18020535

**Published:** 2018-02-10

**Authors:** Jon Lekube, Aitor J. Garrido, Izaskun Garrido, Erlantz Otaola, Javier Maseda

**Affiliations:** 1 Automatic Control Group (ACG), Institute of Research and Development of Processes, Faculty of Engineering, University of the Basque Country (UPV/EHU), 48013 Bilbao, Spain; aitor.garrido@ehu.eus (A.J.G.); izaskun.garrido@ehu.eus (I.G.); erlantz.otaola@idom.com (E.O.); ispmaref@ehu.es (J.M.); 2 Promotion and Subsidies Area, Basque Energy Agency (EVE), Urkixo Zumarkalea, 36, 48011 Bilbao, Spain; 3 Advanced Design and Analysis (ADA), IDOM Consulting, Engineering and Architecture, 48015 Bilbao, Spain

**Keywords:** wave energy, sensing applications, power management, energy harvesting, Wells turbines, Mutriku power plant

## Abstract

Oceans, and particularly waves, offer a huge potential for energy harnessing all over the world. Nevertheless, the performance of current energy converters does not yet allow us to use the wave energy efficiently. However, new control techniques can improve the efficiency of energy converters. In this sense, the plant sensors play a key role within the control scheme, as necessary tools for parameter measuring and monitoring that are then used as control input variables to the feedback loop. Therefore, the aim of this work is to manage the rotational speed control loop in order to optimize the output power. With the help of outward looking sensors, a Maximum Power Point Tracking (MPPT) technique is employed to maximize the system efficiency. Then, the control decisions are based on the pressure drop measured by pressure sensors located along the turbine. A complete wave-to-wire model is developed so as to validate the performance of the proposed control method. For this purpose, a novel sensor-based flow controller is implemented based on the different measured signals. Thus, the performance of the proposed controller has been analyzed and compared with a case of uncontrolled plant. The simulations demonstrate that the flow control-based MPPT strategy is able to increase the output power, and they confirm both the viability and goodness.

## 1. Introduction

Sensors have been widely used in power generation plants during the last centuries. The main role of most of the sensors has been the continuous monitoring of the plant parameters, until recent years. However, some concerns regarding plant security or the need of efficiency improving make sensors essential component within the control system. In addition, renewable energy systems, and in particular marine energy systems, must face new challenges in terms of operation and maintenance (O&M). According to a recent report from the European Union, the annual O&M costs of ocean energy devices can be as high as 5.8% of capital expenditures. In this context, the use of sensors will help mitigate O&M expenses and anticipate failures. 

The potential of marine energy as a source of electricity generation is widely recognized [1,2,3,4,5]. Compared to the approximately 29% currently representing renewable energies in the European energy supply, the challenge of the sector is to increase this percentage to more than 60%. In this sense, wave energy is still situated in the first grade of development, with most devices in TRL 3–4 [6]. The divergence of the technology has slowed down the progress of these devices, not yet being capable of competing with other conventional and unsustainable energy sources [7]. Nevertheless, the first prototypes have shown optimistic results in terms of feasibility and efficiency. 

**Figure 1:**
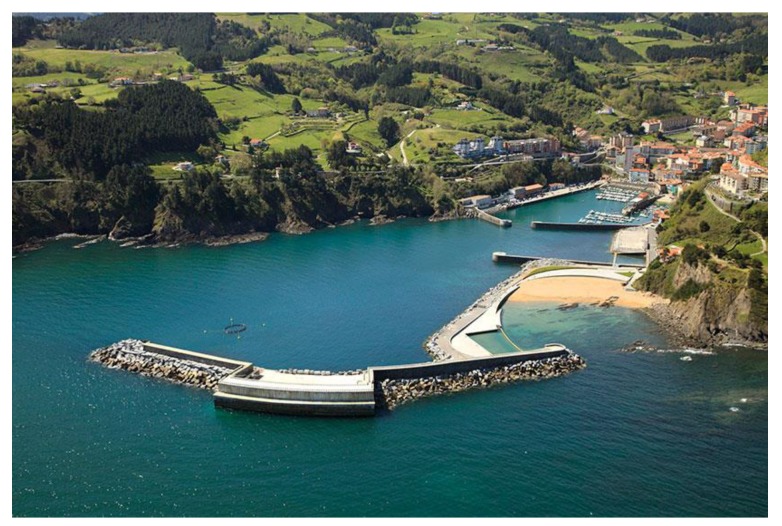
View of the Mutriku Wave Power Plant built at Atlantic coast of the Basque Country [EVE].

In this paper an Oscillating Water Column-based wave energy system is presented. This kind of devices represents a consolidated group within the ocean energy harnessing systems [8,9]. There exist several facilities based on the same principle that have been put into operation in recent years. Figure 1 shows the Mutriku Wave Power Plant, located in the Atlantic coast of the Basque Country. It stands as the first wave power plant composed by more than one turbine and connected to the power grid [10]. It is important to point out that the Atlantic coast offers great possibilities for harnessing wave energy [11,12]. Nowadays the power plant is used as a testing laboratory, where the Basque Energy Agency (EVE) offers the possibility to test large variety of components, for example, turbines or even control methods. The plant is included together with other projects, such as the Biscay Marine Energy Platform (BIMEP), inside the *MaRINET2* project, which joins different testing facilities dedicated to wave energy systems development. Due to its experimental functionality, a large number of sensors are installed along the power plant. Among these sensors stand Oscillating Water Column (OWC) chamber sensors, turbine pressure drop sensors, vibration, temperature or water level sensors.

The main limitation of OWC devices using Wells turbines is imposed by the stalling phenomenon, which produces severe loses in the generated power at low rotational speeds. Therefore, some research has focused on optimizing Wells turbines to avoid negative behavior and to improve their aerodynamics response [13,14,15,16,17]. Nevertheless, novel advanced control methods are needed to make the technology profitable [18,19,20,21,22]. In this context, the Maximum Power Point Tracking (MPPT) control method pursues the objective of optimizing the energy conversion. Although MPPT has already been successfully implemented in other renewable energy systems such as photovoltaic or wind, marine energy systems and in particular OWC devices require a deep study of this control strategy with the aim of adapting it to the new environment [23,24]. 

This article is organized as follows: Section 2 presents a model of OWC devices. In Section 3 the characteristics of sensors and network topology are described. Section 4 implements the control strategy that maximizes the power extraction in OWC devices. The implementations performed for the representative case studies so as to verify the proper operation of the control scheme proposed in this paper were carried out in Section 5. Finally, some conclusions are given in Section 6, that end the manuscript.

## 2. OWC Modeling

In this section, the main parameters taking part in OWC motion, namely airflow velocity, pressure drop and mechanical torque among others, are defined. In this sense, Wells turbine-based OWC device has been modeled by means of the aforementioned parameters and its performance has been described. 

**Figure 2:**
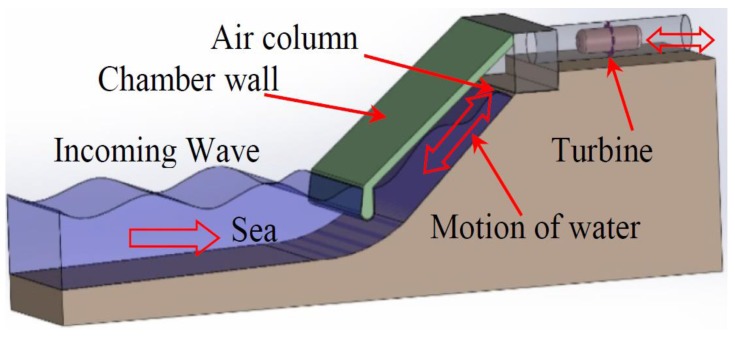
Working principle of Oscillating Water Column (OWC)-based devices.

The working principle of OWC devices is simple [25]. The incoming wave produces air compression within the chamber when the water column is moved towards the top of the chamber. This air-pressure is supposed to produce enough airflow through the turbine duct to make the Wells turbine rotate, as it can be seen in Figure 2. The same action occurs when the wave recedes. The vacuum produced within the OWC chamber during the wave recession produces the same airflow in the opposite direction. 

The airflow velocity crossing the turbine duct can be studied by means of Airy’s theory [26]. There exist adaptations of this theory that consider the combination of regular waves so as to facilitate the study of these systems [27,28,29]. According to the aforementioned process, the airflow velocity at the inlet of the turbine can be estimated as

(1) $$v_{t}(t)=\frac{8awc}{\pi D^{2}}\cdot \sin\frac{\pi l}{cT}\cos\frac{2\pi }{T}t,$$

being *a* the wave amplitude, *T* the period and *c* the speed. Besides, *w* is the width and *l* the length of the OWC chamber and *D* the diameter of the turbine duct.

Despite the bidirectional airflow generated during a wave period, the turbo-generator system is able to keep the same rotational direction by using Wells turbines, as it also occurs with impulse turbines [30]. This kind of turbines, usually known as self-rectifying turbines, always rotate in the same direction regardless the airflow produced by incoming and outgoing waves. Nevertheless, Wells turbines offers very poor efficiencies at low rotational speeds. This behavior is known as stall. Since the turbine response varies at different rotational speeds, there exists a need to define a dimensionless parameter that relates the rotational speed with airflow velocity determined in (1). This parameter is known as flow coefficient, *φ*, and the relationship between both parameters is given by the following equation:

(2) $$\varphi =\frac{v_{t}}{r\cdot \omega_{t}}$$

where $v_{t}$ is the air flow velocity (m/s), *r* is the radius of the turbine blade and $\omega_{t}$ is the turbine rotational speed (rad/s).

**Figure 3:**
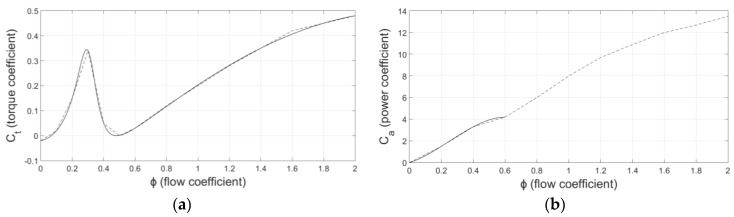
Turbine characteristic curves. (**a**) Torque coefficient vs. flow coefficient; (**b**) Power coefficient vs. flow coefficient. Dashed line: experimental curve. Solid line: approximated curve.

By means of the flow coefficient it may also be estimated the torque coefficient, *C_t_*, and power coefficient, *C_a_*. Both parameters are empirically obtained and stand for own features of Wells turbines. Furthermore, the peak appearing in the torque coefficient is a distinctive feature of Wells turbine which produces the stalling behavior since the torque applied by the turbine drops gradually as the value of the flow coefficient increases above 0.3. *C_t_* and *C_a_* are represented in Figure 3a,b, respectively.

The curves in Figure 3 are composed by different points along the *φ* values according to experimental results. However, they can produce discontinuities in the turbine response. Hence, in order to smooth the response and to facilitate the analytical handling of the model, *C_t_* and *C_a_* have been approximated by the following mathematical expressions [31]:

(3) $$C_{t}=\frac{\sum_{i=0}^{6}p_{i}\cdot \varphi^{i-1}}{\sum_{i=0}^{4}q_{i}\cdot \varphi^{i-1}},$$

where, *p*_1_ = −0.001398, *p*_2_ = 0.01456, *p*_3_ = 0.1408, *p*_4_ = −0.7687, *p*_5_ = 0.9818, *p*_6_ = −0.202, and *q*_1_ = −0.06988, *q*_2_ = −0.3182, *q*_3_ = 0.06089, *q*_4_ = 1, and

(4) $$C_{a}=-25\cdot \varphi^{3}+18.75\cdot \varphi^{2}+4.75\cdot \varphi$$

The responses of (3) and (4) have been represented with solid lines in Figure 3a,b, respectively. In case of (4), it has only been calculated within the operating range so as to simplify the mathematical expression. 

The pressure drop along the turbine can be defined as

(5) $$dP=C_{a}K_{a}\frac{1}{a_{t}}\left(v_{t}^{2}+{\left(r\cdot \omega_{t}\right)}^{2}\right)$$

being $K_{a}=\rho bl_{t}n/2$, where *ρ* is the air density (kg∙m^−3^), *b* is the width (m) and *l_t_* is the length (m) of turbine blades, *n* is the number of blades, and $a_{t}$ is the surface of the turbine duct (m^2^).

Similarly, the mechanical torque developed by the turbine can be written by means of the following expression:

(6) $$T_{t}=C_{t}K_{a}r\left(v_{t}^{2}+{\left(r\cdot \omega_{t}\right)}^{2}\right),$$

where *r* is the turbine tip radius (m).

## 3. Plant Sensors

**Figure 4:**
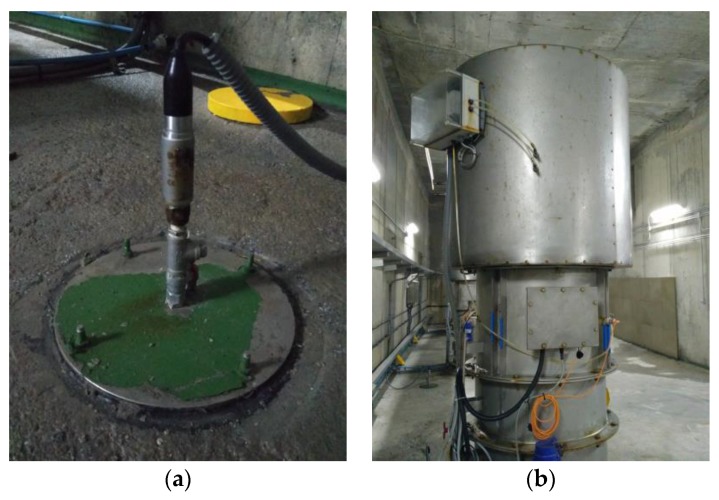
Pressure sensors installed in wave power plant of Mutriku. (**a**) OWC chamber static pressure sensor; (**b**) Wells turbine pressure drop sensor [EVE].

The plant controller usually makes decisions based on the signals coming from different sensors. In this sense, sensors contribute all the necessary inputs to the control system. There exist many sensors that are used in marine environment to measure a wide range of parameters [32,33]. In this particular case, in order to achieve the proposed objectives OWC chamber pressure and the pressure drop along the turbine have been measured by means of pressure sensors. Figure 4 shows two of the many sensors installed along the wave power plant of Mutriku.

The OWC chamber pressure sensor (see Figure 4a) consists of *PTX 7355* type *Druck* sensor. The sensor provides 4–20 mA output signal with 15-bit output resolution. It is able to measure pressures up to +/− 1 bar g, relative pressure, recorded by the plant Supervisory Control and Data Acquisition (SCADA) every 100 ms.

The pressure sensor that measures the pressure drop along the turbo-generator system has two meters, one at the inlet of the turbine and the second at the top of the module, as can be seen in Figure 4b. In addition to this sensor, a static pressure sensor has also been installed at the top of the module. The two pipes may be seen just at the top of the module in Figure 4b. The pressure drop sensor consists of CMR controls P-sensor for low air pressure. It also provides 4–20 mA output signal with 15-bit output resolution. The sensor might measure pressures up to +/− 3 kPa every 100 ms.

**Figure 5:**
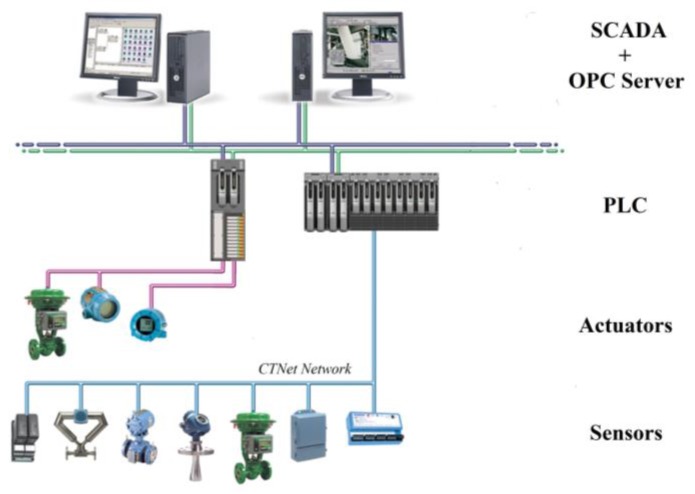
Data collection network topology.

The data from the sensors is collected by a Beckhoff system, which turns both analog signals into fieldbus signals. The connection between Beckhoff and Programmable Logic Controller (PLC) is carried out by CTNet network of *Control Techniques*. CTNet fieldbus is a 5 Mbit Token Ring network that supports peer-to-peer communications. Finally, the SCADA is connected to the PLC by means of an OLE for Process Control (OPC) server/client configuration. Figure 5 shows the network topology used to collect data from sensors.

**Figure 6:**
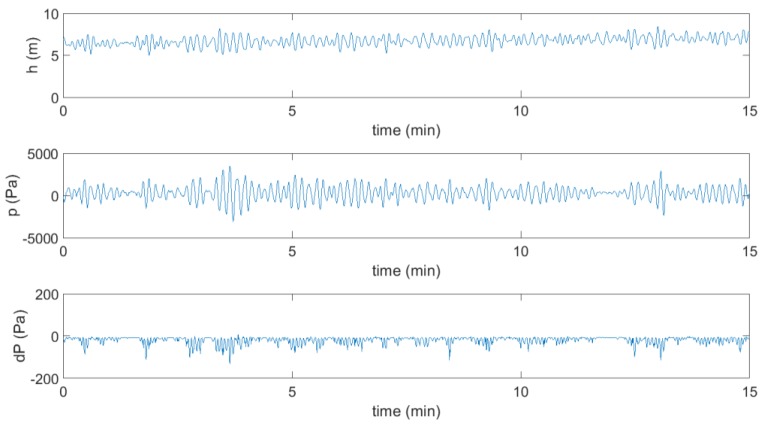
OWC chamber water level and data recorded by pressure sensors installed in wave power plant of Mutriku on 10 October 2017, 6:00 a.m.

Data recorded by both sensors in comparison with the water level within the OWC chamber is presented in Figure 6. This example of data corresponds to a period of 15 min collected on 10 October 2017, starting at 6:00 a.m. 

Previously, a thorough analysis has been carried out by the authors with the aim of developing and establishing the OWC chamber modeling [26]. In that case the model was validated using a 20-min series of wave surface elevation data, collected every 2 h with a sampling period of 0.5 s. Using a Workhorse 600 kHz from Teledyne RDI radar sensor, also known as Acoustic Doppler Current Profiler, high frequency orbital velocities close to the surface were measured. From these measurements, the data used to validate the OWC chamber model were derived. 

## 4. Sensor-Based Flow Control MPPT Strategy

This section describes the control strategy proposed in this manuscript. As has been mentioned before, the objective pursued in this paper consists in the output power maximization by means of a MPPT strategy. This control technique has already been implemented in other renewable energy generation plants, such as wind or solar energy, with successful results. Therefore, in case of wave energy, MPPT should offer similar performance as in already implemented energy systems. However, this strategy must be adapted to wave environment since the behavior of the parameters to be controlled vary slightly from other systems. 

The authors already presented a speed control-based MPPT strategy in [31]. However, in that case the optimum rotational speed was empirically obtained from simulations and curve fitting tools. The current research works provides enough mathematical background to display how the optimum rotational speed is obtained. 

As it happens in most energy systems where the main motion is the rotational movement of the turbo-generator shaft, the performance of the system can steeply drop according to the rotational speed and turbine characteristics. Therefore, in this particular case, MPPT strategy will basically make use of the rotational speed control of the turbo-generator.

However, the expression of the optimum rotational speed must be obtained first. In this sense, Equation (6) has been rewritten so as to obtain the mechanical power expression in terms of the torque ratio to the rotational speed. 

(7) $$P_{t}=C_{t}K_{a}r\omega_{t}\left(v_{t}^{2}+{\left(r\cdot \omega_{t}\right)}^{2}\right).$$

Thus, Equation (7) may be derived with respect to the rotational speed, *ω_t_*:

(8) $$\frac{\partial P_{t}}{\partial \omega_{t}}=K_{a}r\left[\left(v_{t}^{2}+3{\left(r\cdot \omega_{t}\right)}^{2}\right)C_{t}+\left(v_{t}^{2}+{\left(r\cdot \omega_{t}\right)}^{2}\right)\omega_{t}\frac{\partial C_{t}}{\partial \omega_{t}}\right],$$

and the singularities of (8) give the expression of the optimum rotational speed, *ω_opt_*, according to the maximum power point:

(9) $$\omega_{opt}=-\frac{\left(v_{t}^{2}+3{\left(r\cdot \omega_{opt}\right)}^{2}\right)C_{t_{opt}}}{\left(v_{t}^{2}+{\left(r\cdot \omega_{opt}\right)}^{2}\right)\frac{\partial C_{t_{opt}}}{\partial \omega_{t}}},$$

In order to simplify the above equation, Equation (2) has been included in (9), thus obtaining the expression of *ω_opt_* as a function of the optimum flow, *φ_opt_*:

(10) $$\omega_{opt}=-\frac{\left(\varphi_{opt}^{2}+3\right)C_{t_{opt}}}{\left(\varphi_{opt}^{2}+1\right)\frac{\partial C_{t_{opt}}}{\partial \omega_{t}}}.$$

The turbine usually operates in a stalling-free region, that is to say, the flow coefficient should never overcome the value of 0.3 in normal operating conditions. Accordingly, Equation (3) represented in Figure 3a can be simplified within the operating range:

(11) $$C_{t}=-5\varphi^{3}+6\varphi^{2}-0.15\varphi -0.02,$$

whose derivative with respect to *ω_t_* must be obtained:

(12) $$\frac{\partial C_{t}}{\partial \omega_{t}}=\frac{1}{\omega_{t}}\left(15\varphi^{3}-12\varphi^{2}+0.15\varphi \right),$$

and by replacing (11) and (12) in (10) the following expression is obtained:

(13) $$\frac{-10\varphi_{opt}^{5}+6\varphi_{opt}^{4}-5.98\varphi_{opt}^{2}+0.3\varphi_{opt}+0.06}{15\varphi_{opt}^{5}-12\varphi_{opt}^{4}+15.15\varphi_{opt}^{3}-12\varphi_{opt}^{2}+0.15\varphi_{opt}}=0.$$

By solving (13) the value of optimum flow coefficient can be found which, in this particular case, is *φ_opt_* = 0.1294. Moreover, *φ_opt_* only depends on *C_t_*, which represents a design feature of Wells turbines, and thus, this value cannot be employed with other turbines. 

The equation of mechanical power in (7) can be modified by introducing the flow coefficient term according to (2). Thus, *P_t_* can be written as

(14) $$P_{t}=C_{t}K_{a}r^{3}\omega^{3}\left(\varphi^{2}+1\right).$$

Therefore, from (13) and (3) both the flow and torque coefficients are considered unchanging at the maximum power point, and thus, Equation (14) can be simplified as follows:

(15) $$P_{t}=K_{t}\omega^{3},$$

being $K_{t}=C_{t_{opt}}K_{a}r^{3}\left(\varphi_{opt}^{2}+1\right)$.

**Figure 7:**
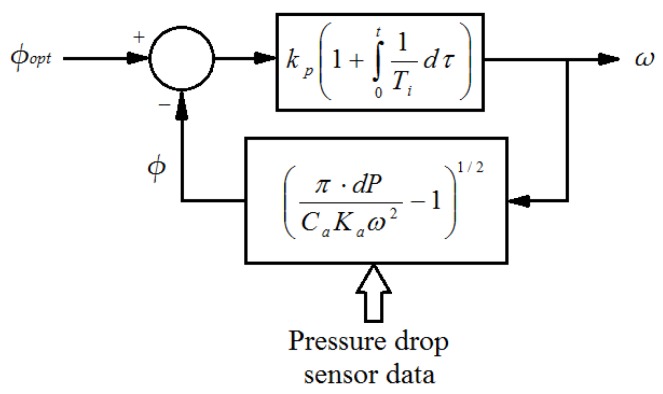
Flow controller control law scheme.

Accordingly, the aim of the flow controller is to keep the operating point around the optimum flow coefficient according to (13). In this sense, the measurement of the current flow coefficient is needed as feedback element. This value is estimated from the measurements taken from the pressure drop sensor (see Section 3). Previously, Equation (5) has been modified taking into account the relation between *φ*, and *v_t_* in (2):

(16) $$\varphi ={\left(\frac{\pi \cdot dP}{C_{a}K_{a}\omega^{2}}-1\right)}^{1/2},$$

where the optimum value of the flow coefficient is known at the maximum power point. The block diagram of the flow controller is shown in Figure 7, where the estimated flow coefficient from (16) is compared with *φ_opt_* obtained from (13). 

Therefore, the closed-loop system may be expressed as

(17) $$\Phi_{opt}(s)=\frac{k_{p}\left(1+\frac{1}{T_{i}s}\right)\cdot \Omega (s)}{1+k_{p}\left(1+\frac{1}{T_{i}s}\right){\left(\frac{\pi \cdot dP(s)}{C_{a}K_{a}\Omega^{2}(s)}-1\right)}^{1/2}},$$

so that, referring to (17) in the time domain one has:

(18) $$\varphi_{opt}(t)=\int_{0}^{t}\frac{k_{p}\left(1+\int_{0}^{t-\tau }\frac{1}{T_{i}}d\tau_{0}\right)}{1+k_{p}{\left(\frac{\pi \cdot dP(t-\tau )}{C_{a}K_{a}\omega^{2}(t-\tau )}\right)}^{1/2}\left(1+\int_{0}^{t-\tau }\frac{1}{T_{i}}d\tau_{0}\right)}\omega (\tau )d\tau .$$

The direct chain flow controller is composed of a proportional-integral control law, which is traditionally used in many control applications. In this case, it provides an adequate performance with minimum tracking error so as to achieve the pursued objective. However, more advanced control systems might replace the aforementioned controller. 

## 5. Results

The proposed control scheme in Figure 7 has been implemented in a wave-to-wire plant model so as to validate the novel MPPT control by means of different simulation case studies.

**Figure 8:**
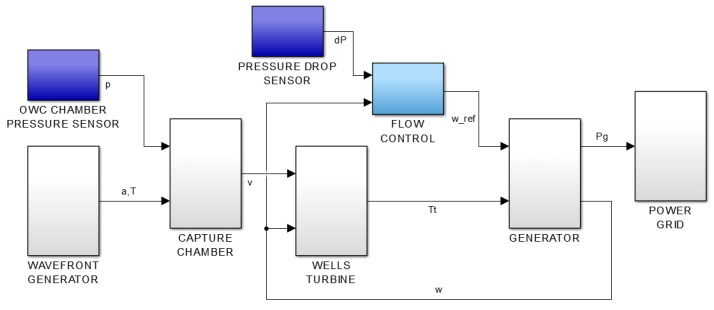
Common control scheme of OWC-based wave power plant with flow control.

For this purpose, as has already been mentioned before, a complete wave-to-wire plant model has been implemented. Figure 8 shows the block diagram of the mentioned plant model, which basically estimates the air flow through the turbine taking into account different wave parameters at the surroundings of the breakwater of Mutriku. Once the value of the air flow velocity has been established, the turbine produces the mechanical torque that makes the generator to rotate. The rotational speed of the turbo-generator is used as closed-loop element, which is necessary in addition to the pressure drop measurement from the sensor to estimate the reference rotational speed. 

In order to operate at the maximum power point, it is first necessary to determine the value of the rotational speed reference for the turbo-generator system. Such a reference speed, *ω_ref_*, is established based on the MPPT strategy. In this sense, the relation between *ω_ref_* and the pressure drop, *dP*, must be optimum so as to guarantee a maximum transmission of the mechanical torque of the turbine to the turbo-generator shaft. Besides, due to the continuous changes in the sea conditions the pressure drop in the turbine varies constantly, as can be seen in Figure 6. Hence, the control system is beholden to set the corresponding *ω_ref_* for each incident wave. 

In order to carry out the experiments, random data regarding the wave amplitudes and periods and corresponding to different sea conditions during five minutes has been used. On the other hand, the parameters used for the OWC chamber, Wells turbine and the induction generator are similar to Mutriku and summarized in Table 1. 

**Figure 9:**
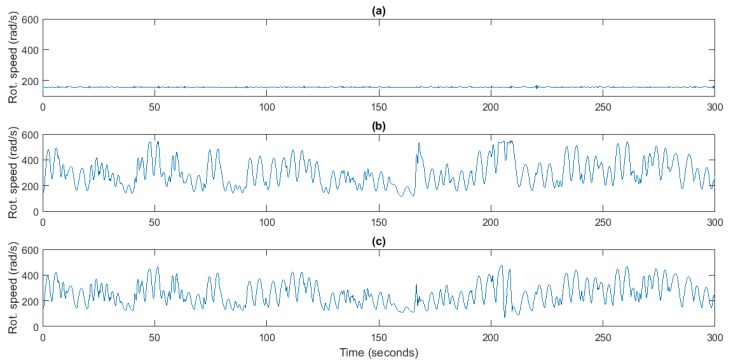
Turbine rotational speed, *ω* (rad/s). (**a**) Uncontrolled plan; (**b**) PI-based speed control; (**c**) Flow Control.

Three case studies have been implemented in order to compare the improvement introduced by the proposed control strategy. In this sense, the first case study considers an uncontrolled plant, where the turbo-generator reaches rotational speeds near the synchronous speed when the generator is connected directly to the grid, as Figure 9 shows. The second case study employs a traditional PI control scheme to implement the speed control strategy as in [31]. To conclude, the third case study uses the flow controller and pressure sensors described in this manuscript so as to implement MPPT control by setting an optimal rotational speed, and thus, maximize the supplied power. Both controllers show a similar performance. Nevertheless, it can be seen that in case of the flow control the rotational speed decreases faster when the wave recedes, so that it improves the efficiency of the converter, as it will be demonstrated in this section. Thereby, it has been possible to analyze both the correct performance of the proposed control strategy and the benefits introduced by it with respect to a PI-based speed controller and an uncontrolled plant. 

**Figure 10:**
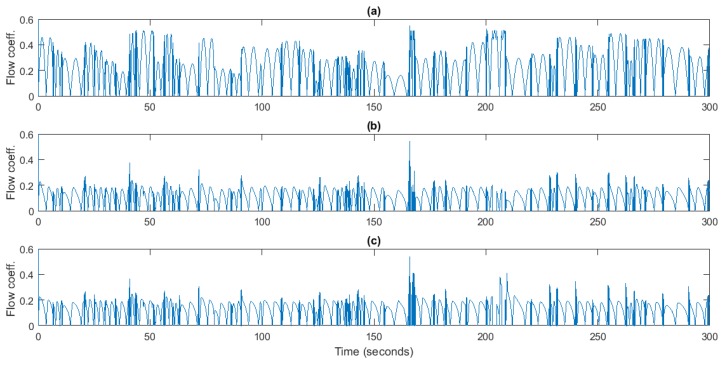
Flow coefficient, *φ*. (**a**) Uncontrolled plant; (**b**) PI-based speed control; (**c**) Flow Control.

Figure 10 shows the comparison of the flow coefficient among the uncontrolled plant, PI-based speed controller and flow controller. In the case of uncontrolled plant, it can be seen that some waves produce values of the flow coefficient beyond the stalling threshold. This means that the turbine steps into stall, so that the supplied power is lower since the efficiency of the turbo-generator drops at these values. On the other hand, in both controllers the flow coefficient remains below the threshold values almost all the time. Therefore, the controller not only guarantees the stalling behavior avoidance, but also improves the power level supplied to the grid.

**Figure 11:**
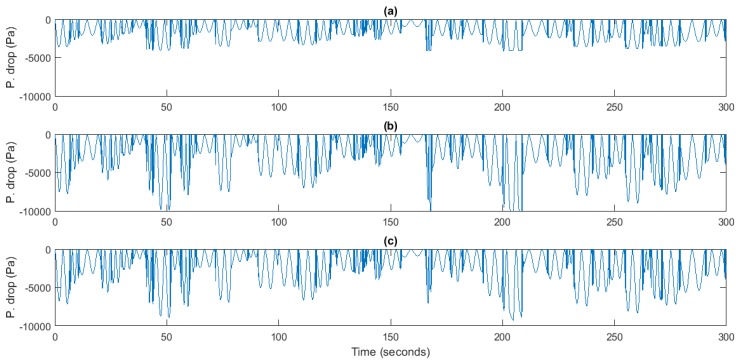
Pressure drop, *dP* (Pa). (**a**) Uncontrolled plant; (**b**) PI-based speed control; (**c**) Flow Control.

Regarding the pressure drop in the turbine, the only conclusion that can be taken is that the controlled case produces larger pressure drops since it simply operates at higher rotational speeds. Figure 11 shows the pressure drop in both controlled and uncontrolled cases.

**Figure 12:**
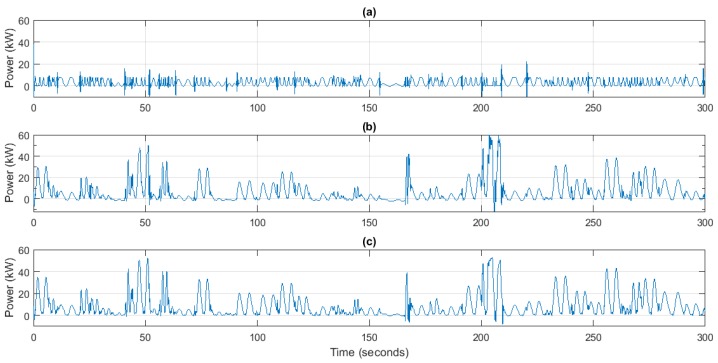
Supplied power, *P* (W). (**a**) Uncontrolled plant; (**b**) PI-based speed control; (**c**) Flow Control.

Finally, the benefits introduced by the proposed controller in terms of generated power have been compared, so that the improvements introduced by the novel control method can be estimated. In this sense, it can be seen in Figure 12 that with the proposed controller the supplied power increases significantly in comparison with the uncontrolled plant. Especially during high waves, 50 kW peaks are obtained in the controlled case, while the uncontrolled case is only able to produce 15 kW, only 30% of available power. 

Comparing the flow controller with the PI-based speed controller, as has been mentioned above, the latter always tries to keep the rotational speed of the generator in the maximum power point, even there is no torque applied in the turbo-generator shaft. The problem in this case is that during the period between the incoming and outgoing waves the airflow through the turbine is practically null. Therefore, since the turbine does not apply any torque, the generator must take energy from the grid to maintain the rotational speed, as can be seen in Figure 12b. This aspect has been considerably improved with the flow controller, since the new control method is able to follow the variations of the pressure drop, and thus the torque, and to decelerate when there no torque is applied. Hence, the efficiency is improved as the generator does not require to be supplied from the power grid.

In terms of power, the maximum power reached by both controllers is similar. Nevertheless, 5 min average power produced by both PI-based speed controller and flow controller has been compared in order to confirm the abovementioned improvement. In this sense, simulations in Figure 12 show that the former controller produces 7.96 kW of average power, while the latter is able to produce 8.82 kW in the same sea conditions. Therefore, by means of the flow control strategy the output power has been increased almost in 1 kW in a 5 min period. This means that the conversion efficiency has been improved by 9.8% in this particular case. 

The simulation results also demonstrate that with the new control scheme the efficiency of the system is improved with respect the controllers implemented by the authors in other works. The previous speed control tried to keep the rotational speed in the maximum power point even when there was no torque applied in the turbo-generator shaft during the period between the incoming and outgoing wave. Therefore, the generator used to take energy from the grid to maintain the rotational speed. This aspect has been considerably improved with the flow controller, since the new control method is able to follow the variations of the pressure drop, and thus the torque, and to decelerate when there is no applied torque. Hence, the efficiency is improved as the generator is not supplied from the power grid.

## 6. Conclusions

In this paper, a new sensor-based flow control MPPT strategy applied to OWC-based wave energy system has been presented. In order to achieve the pursued objective, different pressure sensors have been installed. The first sensor has been used to measure the OWC chamber pressure, whereas the second measures the pressure drop along the turbo-generator. In this sense, a MPPT control method has been implemented so as to maximize the generated power, and thus, to improve the system efficiency. The development of a wave-to-wire plant model has also been carried out and parameterized with measured variables and the real parameters of Mutriku Wave Power Plant. The proposed control scheme has been implemented within the model previously mentioned. The required reference rotational speed of the turbo-generator to maximize the output power has been determined by means of the established flow coefficient control law. The setting of the reference speed is thereby carried out according to the ratio between the measured pressure drop and the rotational speed of the turbine. 

The results demonstrate that the proposed control method can be successfully implemented in OWC-based wave energy device and that the system accurately follows the rotational speed reference established by the pressure sensor-based flow control law so that the efficiency of the system is improved.

## Figures and Tables

**Table 1:** OWC chamber, Wells turbine and generator parameters.

***OWC Chamber and Wells Turbine***		
*w*	Chamber width	4.5 m
*l*	Chamber length	4.3 m
*D*	Air duct diameter	0.75 m
*ρ*	Air density	1.19 kg∙m^−3^
*b*	Blade width	0.21 m
*l_t_*	Blade length	0.165 m
*n*	Number of blades	5
*r*	Turbine radius	0.375 m
***Induction Generator***		
*Snom*	Apparent power	19.892 kVA
*cos ϕ*	Power factor	0.93
*Vnom*	Nominal voltage	560 Vrms
*Fnom*	Nominal frequency	50 Hz
*Rs*	Stator resistance	0.5746 Ω
*Lls*	Stator inductance	746.3 μH
*Rr*	Rotor resistance	0.2974 Ω
*Llr*	Rotor inductancia	4.183 mH
*Lm*	Mutual inductance	129.5 mH
*p*	Number of poles	2
*J*	Inertia	0.55 kg∙m^2^

## Abbreviations

*t*, *c*	Time and propagation speed.
*a*, *T*	Wave amplitude and period.
*w*, *l*	Capture chamber width and length.
*v_t_*, *dP*	Airflow speed and pressure drop.
*ρ*	Air density.
*D*, *r*, *a_t_*	Turbine diameter, radius and area.
*b*, *l_t_*	Blade span and chord.
*n*	Number of blades.
*φ*, *C_a_*, *C_t_*	Flow, power and torque coefficients.
*ω_t_*, *T_t_*, *P_t_*	Rotational speed, torque and power.
Acronyms	
EVE	Ente Vasco de la Energía
OWC	Oscillating Water Column.
MPPT	Maximum Power Point Tracking.
PLC	Programmable Logic Controller.
OPC	OLE for Process Control.
SCADA	Supervisory Control and Data Acquisition.
